# Metabolism of Stone Fruits: Reciprocal Contribution Between Primary Metabolism and Cell Wall

**DOI:** 10.3389/fpls.2020.01054

**Published:** 2020-07-09

**Authors:** Monica Canton, María F. Drincovich, María V. Lara, Giannina Vizzotto, Robert P. Walker, Franco Famiani, Claudio Bonghi

**Affiliations:** ^1^ Department of Agronomy, Food, Natural Resources, Animals and Environment, University of Padova Agripolis, Legnaro, Italy; ^2^ Facultad de Ciencias Bioquímicas y Farmacéuticas, Centro de Estudios Fotosintéticos y Bioquímicos, Consejo Nacional de Investigaciones Científicas y Técnicas, Universidad Nacional de Rosario, Rosario, Argentina; ^3^ Department of Agricultural, Food, Environmental, and Animal Sciences, University of Udine, Udine, Italy; ^4^ Dipartimento di Scienze Agrarie, Alimentari e Ambientali, Università degli Studi di Perugia, Perugia, Italy

**Keywords:** endocarp, ascorbic acid, cyanogenic compounds, lignin, cell wall turnover

## Abstract

Cell wall turnover and modification in its composition are key factors during stone fruit development and patterning. Changes in cell wall disassembly and reassembly are essential for fruit growth and ripening. Modifications in cell wall composition, resulting in the formation of secondary cell walls, are necessary for producing the most distinctive trait of drupes: the lignified endocarp. The contribution of primary metabolism to cell wall synthesis has been investigated in detail, while the knowledge on the contribution of the cell wall to primary metabolites and related processes is still fragmented. In this review, starting from peculiarities of cell wall of drupes cells (in mesocarp and endocarp layers), we discuss the structure and composition of cell wall, processes related to its modification and contribution to the synthesis of primary metabolites. In particular, our attention has been focused on the ascorbate synthesis cell wall-related and on the potential role of cyanogenic compounds in the deposition of the secondary cell wall.

## Introduction

Cell wall metabolism is an integral part of the primary metabolism since the cell wall is the primary carbon sink in many plant tissues. The majority of C is stored into the cell wall polysaccharides making this cellular component the most important biomass on earth. In addition to polysaccharides (cellulose, hemicelluloses, and pectins), cell walls are composed by other, quantitatively minor but functionally important components, such as polyphenols and proteins, many of which are glycosylated (Somerville et al., 2004). Plant cell walls composition is highly variable not only among species but also within an individual plant at both the tissue and cell levels (Zhang et al., 2014). Moreover, cell walls are classified as primary walls, which are surrounding the cell driving its growth and consequently also its morphology, and secondary walls, whose rigidity and strength is necessary to fulfill specialized cell functions (Somerville et al., 2004). In stone fruits (or drupes), cell wall changes resulting in the formation of secondary cell walls are particularly important because they are necessary for producing the most distinctive trait of drupes: the lignified endocarp.

## Primary Metabolism and Primary Cell Wall

### Primary Cell Wall Composition and Its Metabolism During Drupe Growth and Ripening

Similarly to other fleshy fruit, an active cell wall turnover is essential for a correct fruit development and ripening in *Prunus* spp. More than 50 cell wall-related genes encoding for lignocellulose-degrading enzymes and nonenzymatic protein (e.g., expansins, EXP) (Goulao and Oliveira, 2008; Mercado et al., 2011; Gapper et al., 2013), as well as components of subcellular structures (e.g. cytoskeleton), have been claimed to be involved in the cell wall turnover (Bashline et al., 2014). Drupe growth is the result of coordinated cell divisions and expansion processes in which cell turgor pressure, cell wall biosynthesis and its remodeling play a fundamental role. During fruit ripening, cell wall disassembly, combined with a decrease of cell turgor pressure, is the main process responsible for fruit softening. In this context, before discussing how the cell wall changes during fruit development and ripening and its contribution to primary metabolism, it is necessary to provide some information about the cell wall composition of *Prunus* spp. fruit.

Parenchyma cells with a thin primary wall are the main cell type present in fleshy fruit tissues. In dicotyledonous plants, the primary cell wall is composed roughly by equal part, ranging around 30%, of cellulose, hemicelluloses, and pectins, plus 1–10% of structural proteins. Fruit cell walls have also high content of water (Posé et al., 2018).

A detailed description of each component is not the main goal of this review, however, here it will be summarized the newest results on cell wall component reached by employing the Atomic Force Microscopy (AFM), an imaging tool for studying food macromolecules and colloids (Gunning and Morris, 2017). The advantage of AFM, in comparison to techniques based on high-resolution scanning electron microscopy, is the avoidance of cell wall polymers coalescing because it is not necessary to dehydrate the sample before the analysis. Thanks to this characteristic, AFM, in the last decade, has opened a new vision of structural features of cell wall components, particularly polysaccharides.

Among polysaccharides, cellulose is the main component of the primary cell wall. It is composed of a repetitive unit formed by residues of β-(1–4) linked d-glucose. These are arranged in fibrils. By using AFM, Niimura et al. (2010) demonstrated that cellulose microfibrils present in peach fruit are ultra-thin (diameter ranging between 1 and 2 nm). Based on this characteristic, peach cellulose nanofibrils can be classified as dietary fiber, and therefore, they can heavily contribute to the nutritional value of peach fruit.

Xyloglucan (XyG) is the most abundant hemicellulosic component, this polymer is embedded in an amorphous pectin matrix composed of polygalacturonides together with other less abundant components, such as phenols, structural proteins, enzymes, and a variety of receptors and sensors (Goulao and Oliveira, 2008; Posé et al., 2018). Differences in the thickness of hemicellulose chains have been related to differences in fruit texture. Chen et al. (2009) reported that tick hemicellulose chains were more abundant in cherries with crisp texture than the softer ones.

Pectin is a heterogeneous polysaccharide mainly composed of d-galacturonic acid (GalA) (Fishman et al., 2004; Yang et al., 2009; Wang et al., 2018). Visualization of the pectin sample isolated from the fruit by AFM has confirmed the great nano-structure heterogeneity. In peach, plum, and apricot fruit, pectins are naturally aggregated in large (1–3 nm) and branched fiber (Yang et al., 2009; Liu et al., 2017; Mierczynska et al., 2017). The presence of long pectin chains (longer than 1000 nm) is usually associated with a consistent texture/relatively high firmness, while fruits with thinner and shorter pectin chains (e.g. strawberry and tomato) undergo to a rapid softening. A reduction of the complexity of pectin nanostructure occurs during the fruit softening of Prunus ssp., as observed by Zhang et al. (2008) in Chinese cherry. In this study, differences in the structure of chelated pectins that were presently moving from the first growth phases to ripening have been detected by AFM, being pectins from unripe fruit longer and wider than those from ripe fruit.

Modifications of cell wall composition and structure are the foundation of changes in fruit firmness and texture during ripening. Some changes in cell wall components ultrastructure appear to be common (e.g. the hemicellulose depolymerization), but others occur in a specie-specific manner or are totally absent. For example, a slightly decrease of cellulose content occurs during ripening in most fruits, although this event is often uncoupled with the increase of crystalline cellulose in ripe fruit (Posé et al., 2018). On the other hand, although in cell wall galactose (Gal) and arabinose (Ara) level declines in ripe fruit of most species, Gal and Ara loss does not take place in plum, and Ara reduction is minor or absent in apricot. In peach, XyG depolymerization is among early events during softening (Brummell et al., 2004; Posé et al., 2018), while other fleshy fruit such as apple (Percy et al., 1997) the ripening proceeds in absence of the XyG depolymerization or this event is cultivar-dependent as in strawberry (Rosli et al., 2004).

Fruit softening is a very important event during ripening (Payasi et al., 2009) and it, primarily, results from both the decline in cell wall strength and cell-to-cell adhesion caused by modification of mechanical properties of cell wall and the depolymerization of pectins forming the middle lamella, respectively. These modifications together with the turgor pressure decline, that is associated with an increase in the concentration of apoplastic solutes (Wada et al., 2008), lead to fruit juiciness and texture softening (Toivonen and Brummell, 2008).

Pectins are the cell wall components showing the highest structural modifications during ripening; however, their role in fruit ﬁrmness and softening is still extremely controversial (Paniagua et al., 2014). These changes are an early solubilization and loss of neutral side chain, and, later on, a depolymerization mainly by polygalacturonases (PGases) (Goulao, 2010). Pectin solubilization may occur when cohesion of pectin molecules is weakened by the loss of neutral sugars in the form of neutral galactose-rich side-chains of rhamnogalacturonans 1 (RG-I). Neutral side chains from RG-I might aggregate pectins either by physical interaction with other cell wall polymers or by binding to hemicellulose and cellulose (Zykwinska et al., 2008). In the Colorless non-ripening (Cnr) tomato fruit, the deposition of (1→5)-α-l-arabinan, which is the constituent the branched sidechain of RG-I, is impaired resulting in a reduced length and the low esterification degree of pectin (Orfila et al., 2001). The consequence of the altered Cnr fruit pectins structure is the lack of pericarp swelling and the presence of large intercellular space in the inner pericarp in comparison with the wild type fruit. Loss of cell adhesion has been also observed between cells of leaf parenchyma and abscission zone of transgenic apple plants overexpressing a PG gene. The overexpression of PG leads to a formation of pectins with shorter chains in comparison to those observed in the wild-type plants (Atkinson et al., 2002). These observations confirm that the cell adhesion strength is related to the composition of pectins forming the middle lamella (Jarvis, 1984). Later on, a more detailed analysis of cell wall microstructure of Cnr fruit parenchyma cells located in the interface with neighboring cells highlighted the presence of xylan and xyloglucan (Ordaz-Ortiz et al., 2009).This result indicates that hemicellulose polymers are structural cell wall component involved in the cell adhesion/cell separation process. Depolymerization of pectins during ripening is largely due to result of a sequential and coordinated action of several pectin-metabolizing enzymes such as PGases, pectinmethylesterases (PME) and pectinlyases (PL) (Brummell et al., 2004; Morgutti et al., 2006). Among PGs, endo-polygalacturonase (endo-PG) plays a central role in the depolymerization of cell wall pectins of peach fruit; however, endo-PG is essential for the achievement of a melting flesh (MF) fruit texture, due to the loss of cell adhesion, but not for reducing fruit firmness. The localization of endo-PG isoforms at the middle lamella of the cell wall of MF fruit supports this role (Morgutti et al., 2006). In addition, in non-MF (NMF) no endo-PG was detected and consequentially no loss of cell adhesion was observed. On the basis of these observations, the role of endo-PG activity on the reduction of fruit firmness has been debunked because of NMF peaches are able to soften and, at the same time, change of symplast/apoplast water status has been suggested as the main mechanism through which peach fruit firmness is regulated. A re-thinking of the main role of pectin depolymerization in the fruit softening (Wang et al., 2018) has been proposed for other fleshy fruits including strawberry and apple also on the basis of observation carried out after the silencing of PL (Posé et al., 2013) and PG (Atkinson et al., 2012), respectively. In both species the silencing of pectolitic enzymes lead an increase in cell-to-cell adhesion together with slight depolymerization of pectins and an improvement of fruit firmness and textural proprieties, without affecting other fruit quality traits.

Level of cell wall hydrolases change accordingly with the variation of the transcription of the corresponding genes of these enzymes (particularly PGases and PL) in ripening fruit, as pointed out by several transcriptome studies (Trainotti et al., 2006; Pan et al., 2016; Pei et al., 2019). In ripening peaches, Pei et al., 2019 also reported the up-regulation of xyloglucan endotransglucosylase/hydrolases (XTHs), responsible for the reduction of mass of wall-bound xyloglucans and consequently able to increase the cell wall extensibility. Worthy of note is the fact that the action of XTHs is induced by xyloglucan oligosaccharides (XGOs) and that, during peach fruit ripening, Pei et al. (2019) observed the down-regulation of two esterase/lipase proteins (GELPs) known for their action against XGOs.

### Contribution of Cell Wall Disassembly to Primary Metabolites in Ripening Fruit

**Figure 1:**
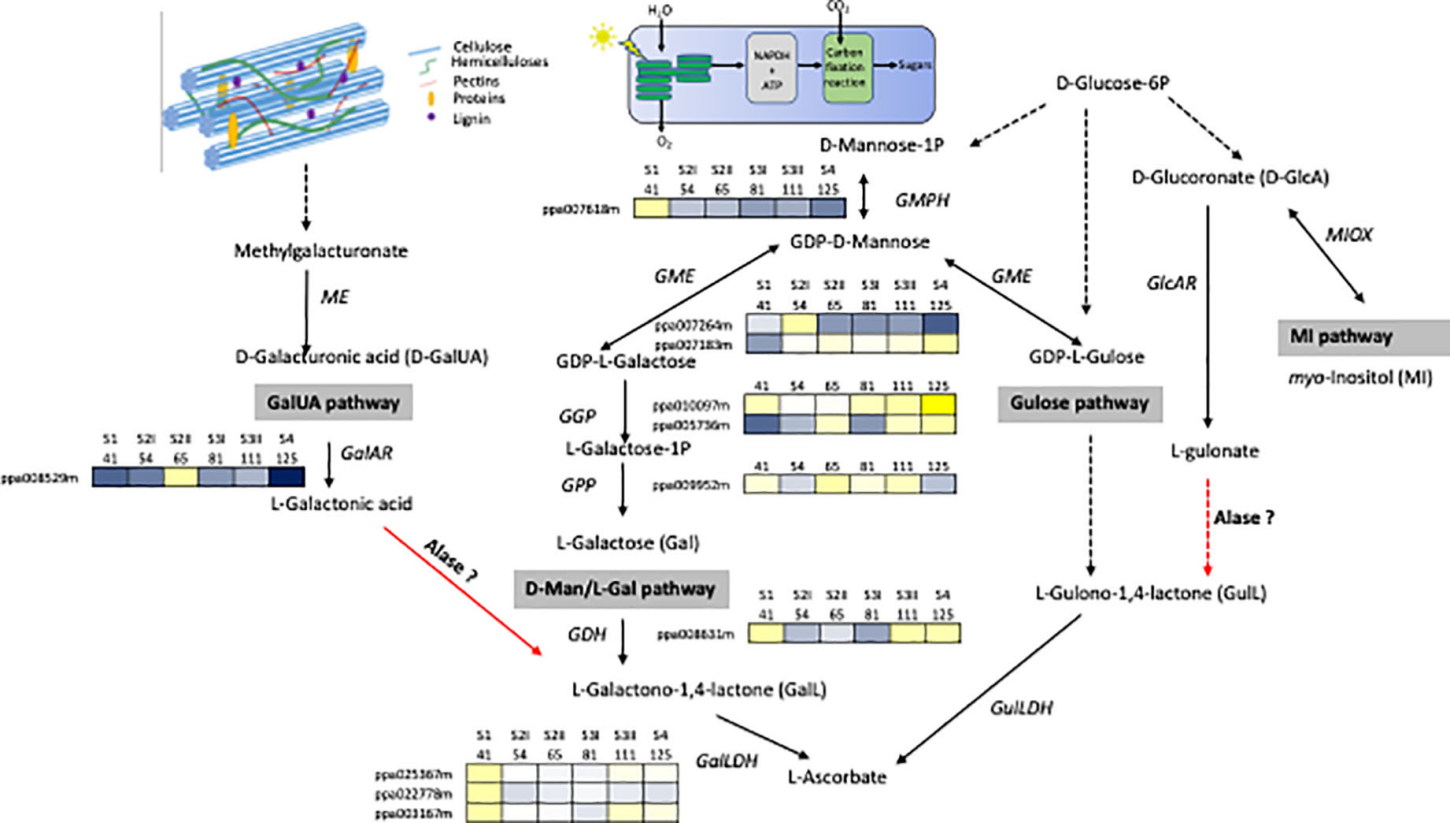
Network for the biosynthesis of AsA. The four possible pathways include the d-galacturonic acid (GalUA), l-galactose (Gal), l-gulose, and myo-inositol (MI). Enzymes catalyzing reaction are Alase, aldonolactonase; GalLDH, l-galactono-1,4-lactone dehydrogenase; GalAR, d-galacturonate reductase; GDH, l-galactose dehydrogenase; GlcAR, d-glucuronate reductase; GME, GDP-d-mannose -3′,5′-epimerase; GulLDH, l-gulono-1,4-lactone dehydrogenase; ME, methyl esterase, MIOX, myo-inositol oxygenase; GMPH, GDP-d-mannose pyrophosphorylase; GGP, GDP-l-galactose phosphorylase; GPP, l-galactose 1-P phosphatase. Broken arrows show more than one enzymatic reaction step. Red arrows indicate steps still missing in higher plants. Transcripts profiles of GMPH, GME, GGP, GPP, GDH, GalLDH, GalAR genes during peach fruit development (cv Fantasia) have been retrieved from Gene Expression Omnibus (GEO) database under accession number GSE71561. Each gene is identified by the transcript code (peach genome version 1, see at https://www.rosaceae.org/organism/24333). Transcript levels significantly decreased were displayed in blue, while transcript levels significantly increased were displayed in yellow. The brightness of each color corresponded to the magnitude of the difference when compared with the average value.

Cell wall degradation during ripening contributes substantially to the change level of primary metabolites fundamental for the human diet. It is high probable, that the quantity of these metabolites is strictly related to the composition and structure of polymers of the primary cell walls and the middle lamella as well as the disassembly mechanisms that can differ among species and within them among cultivars. However, at the moment, studies on the contribution of cell wall disassembly have been focused on the impact of pectin depolymerization on the ascorbate level (AsA, vitamin C). The biosynthetic pathway of AsA in plants can be represented with a complex network in which different pathways are converging: d-mannose/l-galactose (d-Man/l-Gal) (Wheeler et al., 1998), l-glucose (Wolucka and Van Montagu, 2003), myo-inositol (Lorence et al., 2004) and d-galacturonic acid (d-GalUA) (Agius et al., 2003), which is a component of pectins (**Figure 1**). Which pathway predominates is dependent on the species, tissue and stage of development (Walker and Famiani, 2018). The degradation of pectins releases methyl-galacturonate (Smirnoff et al., 2011), which is then converted into d-GalUA by a (pectin) methyl esterase (Paciolla et al., 2019) and successively into l-galactonic acid by d-galacturonate reductase (GalAR), firstly isolated in strawberry (Agius et al., 2003). An aldonolactonase (Alase), up to now isolated and well characterized only in Euglena (Ishikawa et al., 2008), converts l-galactonic acid into l-galactono-1,4-lactone, which is the last precursor of vitamin C (**Figure 1**). The d-Man/l-Gal pathway has been reported for many fruit-bearing plants, such as kiwifruits, acerola, peach, and tomato (Badejo et al., 2009; Bulley et al., 2009; Imai et al., 2009; Ioannidi et al., 2009), but these evidences are only clear for developing fruits, while there is still obscure how the AsA pool size is controlled during fruit maturation. A study carried out on microtomato fruit, based on feeding experiment with potential AsA precursors, suggests that it could be activated a switch from d-Mann/l-Gal to Ga1UA pathway moving from immature to ripe fruit (Badejo et al., 2012). In peach, genes involved in the conversion of sugar pool into l-Galactose were showing different expression pattern although the majority of them were highly expressed in the early phases of fruit development (**Figure 1**).The expression level of l-galactose dehydrogenase (GDH) and l-galactono-1,4-lactone dehydrogenase (GalLDH), the most important genes involved in the d-Man/l-Gal pathway were showing a biphasic expression profile with maximal at early stage and, at lower extent, during ripening phase. Transcript accumulation of GDH and GalLDH and AsA content, expressed per gram fresh weight basis, were related in the early period of fruit development, whereas this relationship was less evident in the last phase of fruit development in which AsA was at the lowest content (Imai et al., 2009). This result opens the possibility that in peach, as suggested in tomato, a switch between the AsA biosynthetic pathways can occur at ripening. Experiment feeding with d-GalUA of peach ripe fruit induced an increase of in the reduced form of AsA (Imai et al., 2009). At the moment, for GalUA pathway it is available only the expression of GalAR that shows the lowest expression at ripe stage, no information is available for Alase. Investigations are necessary to demonstrate if this mechanism may be present in peach or other Prunus species. For this goal, the major constrain is the lack of identified orthologs to the *Euglena* Aldolactonase in higher plants. However, a quantitative trait locus (QTL) analysis allowed the identification of five regions and two of them included genes annotated with terms related to the known d-Man/l-Gal and AsA/glutathione pathways (Stevens et al., 2007). Based on this result, it is probable that genes encoding the unidentified enzymes for the d-GalA pathway could reside in the rest of the candidate loci.

Partial cell wall degradation at ripening also leads to a massive release of sugars, which in plants are recycled for providing energy and building units for a large number of processes, including synthesis of protein and sugar accumulation. An indirect evidence of this causal relationship was obtained in fruit of transgenic plant of tomato in which PME transcripts were almost abolished by an antisense approach (Tieman et al., 1992). In particular, fruits from transgenic tomato plant were richer in soluble neutral sugar (sucrose) than wild-type fruits.

## Primary Metabolism and Formation of The Lignified Endocarp

Both the end-products and intermediates of primary metabolism are precursors of secondary metabolites (Douglas, 1996). The amino acid phenylalanine (Phe) is an example of the interconnection between primary and secondary metabolism because Phe can be a protein building block or a precursor of lignin, a secondary metabolite essential for plant growth, development, and defense (Pascual et al., 2016). Here, we first describe the structural characteristics of a lignified endocarp and then we analyze the role of primary metabolism in its formation.

### Structural Characteristics of Drupe Pit

The lignified endocarp (or pit) is a distinctive trait of mature drupe fruits, but its formation occurs relatively early in fruit development, and its subsequent lignification takes place in phase II of the double sigmoid fruit-growth curve, in which the mesocarp growth is suspended (Bonghi et al., 2011; Rapoport et al., 2013). This alternate pattern of growth between the different fruit tissues suggests the presence of cyclic events of competition for assimilates among fruit tissues and seed (Bollard, 1970; Opara, 2000). During stage II, when the endocarp is lignifying, the endosperm, throughout the absorption of nucellus, grows largely and later on, the embryo development is sustained by metabolites stored in the endosperm (Bassi and Ryugo, 1990; Ognjanov et al., 1995; Walker et al., 2011; Famiani et al., 2012). Therefore, one view is that the cost of embryo growth and endocarp lignification, in terms of assimilates, results in a temporary cessation of mesocarp growth. However, it is also possible that the temporary cessation in growth is brought about either largely or in part by genetic information that controls development (Pavel and DeJong, 1993b). The pit hardening is a progressive event as suggested by both anatomical observations and chemical analysis of lignin deposition. In the olive fruit, the time occurring for pit hardening takes, after bloom, a period ranging from 5 weeks up to 3 months (Hartmann, 1949; Lavee, 1986). In nectarine, the evaluation of force required to crush the endocarp pointed out that, although sclerification occurred slowly, the degree of hardness increased sharply around 12 to 13 weeks post-flowering (King et al., 1987). However, in peaches, the timing of this event is more related to the ripening time and shows large differences among early, mid, and late ripening peach cultivars (Pavel and DeJong, 1993a; Yamaguchi et al., 2002). In particular in early ripening peach and plums cultivars, the second exponential growth phase (Stage III) of fruit starts before the endocarp has completely lignified (Kritzinger et al., 2017). Additional factors in the variation of pit hardening timing are the tree water status (Rapoport et al., 2004; Lavee et al., 2007) and temperature (Dardick and Callahan, 2014; Souza et al., 2019).

Endocarp expansion ceases with the deposition of a thick, lignified secondary wall in endocarp cells (King, 1938; Dardick et al., 2010; Rapoport, 2010). In peaches, it has been reported that the onset of wall thickening and lignification of endocarp cells starts at the stylar end (Hayama et al., 2006; Dardick et al., 2010) and, then, proceeds toward the stem end of the fruit (Sterling, 1953; Lilien-Kipnis and Lavee, 1971). The presence of lignin in the peach endocarp was firstly reported by Ryugo (1963) in the early 1960s. This observation has been supported by a subsequent study in which the accumulation of lignin precursors (phenol bodies) was observed in endocarp cells (Masia et al., 1992). The lignin biosynthesis results from a sequential involvement of phenoloxidase, peroxidase, and laccases (Alba et al., 2000; Dardick et al., 2010). In peach endocarp cells, phenoloxidase was detected mainly in the ionically bound cell wall protein fraction suggesting its role in the polymerization of lignin precursor. This localization could suggest the engagement of this enzyme in the early changes of cell wall undergoing lignification, such as the polymerization reactions of oligolignols, occurring at the end of the first stage of development. Peroxidase and laccases seem more related to the late stage of sclerefication process by aiding the cross-coupling between the growing polymers. To support this vision, the activity of peroxidase and laccase increases concurrently with the rise of lignin content detected during the second stage of fruit development.

Additional information on lignin deposition in cell wall during drupe development and patterning have obtained from a Slow Ripening (SR) peach phenotype in which fruit development is apparently stopped during the stage III (Bonghi et al., 2011), and the flesh shows a very slow rate of softening accompanied by a low level of ethylene (Brecht and Kader, 1984). A metabolomic analysis of SR fruit, pointed out a strong accumulation of phenylpropanoids (in particular lignin and its precursors) in the mesocarp paralleled by the expression of phenylpropanoids biosynthetic genes (Botton et al., 2016). This evidence, together with microscopic analysis, suggests that SR mesocarp behaves like an endocarp. The comparison of the expression profile of genes responsible for endocarp identity in SR and Fantasia allowed the identification of an additional regulator of endocarp lignification named FLESHY, similar to Arabidopsis HECATE3 (Botton et al., 2016). In SR fruit, FLESHY shows a transient increase in the mesocarp while remaining at a very low level in Fantasia mesocarp. Therefore, FLESHY has been claimed to play a crucial role in determining the fruit tissue patterning of the peach fruit.

In *Prunus* spp, there is a strong variability of endocarp phenotypes, the most part of them have been obtained by using traditional breeding. Dardick and Callahan (2014), reported that almond shells were found to differ according to endocarp thickness, hardness, and bitterness. The seed of some peach, apricot, and plum varieties is easily exposed to pests and diseases as a consequence of the unsealed endocarp. This defect, named “split pit”, is the result of a down-regulation of phenylpropanoid biosynthetic genes (Zhang et al., 2017). Environmental conditions (Engin et al., 2010), cultivation practices (Claypool et al., 1972) and the ripening time, play a role in the development of split pit. In particular, early maturing peach and plum cultivars are usually more susceptible to stone splitting, because their stones do not harden properly for resisting the growing forces of the rapidly expanding fruit flesh (Tani et al., 2009).

A natural phenotype isolated in a wild-type population of plum was called “Stoneless” for its incomplete development of the endocarp layer that results in a partially naked seed (Callahan et al., 2009). The stoneless phenotype is strongly affected by the environment conditions since a complete endocarp can develop in years with hot spring temperatures, while in cooler years very little stone is present. The absence of endocarp tissue suggests that this mutant does not contain a complete endocarp layer (Dardick and Callahan, 2014).

### Contribution of Primary Metabolism to the Formation of a Lignified Endocarp

**Figure 2:**
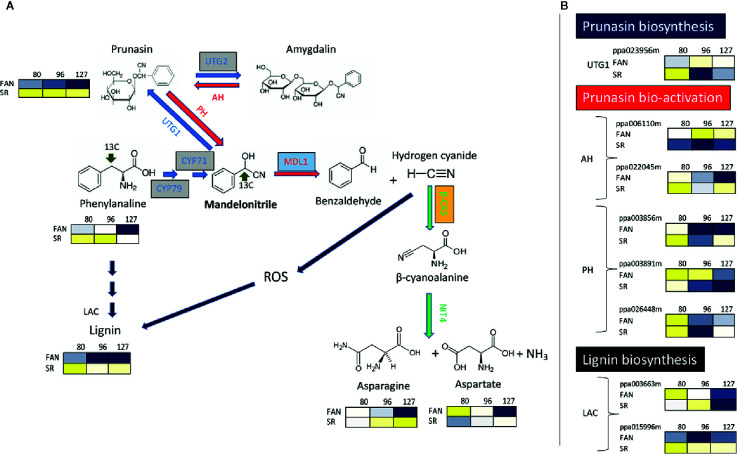
The metabolic pathways for synthesis, bio-activation, and detoxification of the cyanogenic glucosides prunasin and amygdalin in the peach mesocarp of Fantasia (FAN) and slow ripening (SR). **(A)** Biosynthetic enzymes/genes (blue arrows) are: CYP79 and CYP71 (Cyt P450 monooxygenases); UGT1 (UDPG-mandelonitrile glucosyltransferase); and UGT2 (UDPG-prunasin glucosyltransferase). Bio-activation enzymes/genes (red arrows) are: AH (Amygdalin hydrolase); PH (prunasin hydrolase); MDL1 (mandelonitrile lyase). Detoxification enzymes/genes (green arrows) are: β-CAS (β-cyanoalanine synthase), NIT (Nitrilase 4). Lignin biosynthetic enzymes/genes (black arrows) LAC (Laccases). Levels of Phenylalanine, Prunasin, Asparagine, Aspartate at three fruit developmental stages (80 DAFB, late S2; 96 DAFB, early S3; 127 DAFB, pre-climacteric S4) assessed in both the genotypes (Fantasia= FAN, and slow ripening= SR). Metabolites significantly decreased were displayed in blue, while metabolites significantly increased were displayed in yellow. The brightness of each color corresponded to the magnitude of the difference when compared with average value. **(B)** Expression profile of genes involved in biosynthesis and bio-activation of cyanide glucosides, and in the last step lignin biosynthesis evaluated in FA and SR mesocarp at the three developmental stage reported in A. Each gene is identified by the transcript code (peach genome version 1, see at https://www.rosaceae.org/organism/24333). Transcript levels significantly decreased were displayed in blue, while transcript levels significantly increased were displayed in yellow. The brightness of each color corresponded to the magnitude of the difference when compared with the average value. Metabolites and transcripts level were retrieved from Botton et al. (2016). CYP79, CYP71, and UTG2 genes, boxed in grey, have been characterized only in almond seeds and their putative orthologs have been identified in peach (Thodberg et al., 2018). Transcripts of MALD1, boxed in light blue, have been detected in the endocarp layer of apricots (Zhang et al., 2017). Enzyme activity of β-CAS, boxed in orange, has been determined in peach endocarp (Hu et al., 2012; Rodriguez et al., 2019).

It has been reported that the activity of the most enzymes involved in primary metabolism are repressed during lignin and flavonoid biosynthesis in the endocarp layer (Dardick et al., 2010). However, this contrasts with the observation by Hu et al. (2012), which found the lignin content is positively related with the Pyruvate Dehydrogenase (PDH E1α) protein level, a well-characterized enzyme complex that links two of the most important metabolic pathways of primary metabolism: glycolysis and TCA cycle (Tovar-Méndez et al., 2003). In addition, another PDH gene (a sub-unit called PDH E1β) has been identified as a member belong to a regulon that is induced in correspondence of lignin deposition in the peach endocarp layer (Dardick et al., 2010). One interpretation of these conflicting observations is that the expression of enzyme abundance was on a per DW basis and during lignification there is a large increase in the DW content of the tissue; and thus the decrease is just a dilution effect (Famiani et al., 2015). In support of this in both plum and cherry endocarp, a large number of enzymes involved in primary metabolism are abundant (or actually increase in abundance) on a per FW basis during lignification (Walker et al., 2011; Famiani et al., 2012). On the other hand, the relevant impact of endocarp lignification on fruit primary metabolism is suggested by the rerouting of several primary metabolites toward lignin biosynthesis. A decrease of protein synthesis has been observed during the very early phase of peach development, which follows the use of free amino acids as substrates for the synthesis of phenylpropanoids required for endocarp lignification (Lombardo et al., 2011; Rodriguez et al., 2019). Amino acids, phenylalanine in particular, are also precursors of cyanide glucosides such as prunasin, which are nitrogen-containing secondary metabolites that strongly accumulate in Prunus fruit. Cyanide glucosides have the ability to produce highly toxic hydrogen cyanide (HCN) when cleaved by mandelonitrile lyase. Differential expression of a putative mandelonitrile lyase gene has been observed in apricots having endocarp with different thicknesses and lignin content (Zhang et al., 2017). Previous reports have demonstrated that HCN can generate reactive oxygen species (ROS) (Oracz et al., 2009). Accumulation of ROS has been observed in tissues, including endocarp, undergoing lignification (Liu et al., 2017). The detoxification of hydrogen cyanide, and consequently a potential reduction of ROS, is catalyzed by β-cyanoalanine synthase (Blumenthal et al., 1968; Machingura et al., 2016). It has been reported that during early phases of fruit development (up to 59 DAFB corresponding to S2) β-cyanoalanine synthase protein level shows a decreasing trend (Hu et al., 2012) or it almost stable (Rodriguez et al., 2019) during the lignification of endocarp, while it is increasing in the mesocarp (Hu et al., 2012; Rodriguez et al., 2019). These pieces of evidence suggest that endocarp lignification is accompanied by an increase of ROS precursors due to an increase of cyanide glucosides and reduced or stable detoxification of the action of hydrogen cyanide. To support this view, in the lignin-rich mesocarp of SR peach mutant it was observed a higher level of prunasin, paralleled by the accumulation of genes involved in its biosynthesis, in comparison to wild type peaches (**Figure 2**; Botton et al., 2016).

In addition, the interconversion between nucleotide sugars is affected by the lignification process on fruit primary metabolism. In peach, xylans are the most important component of hemicelluloses in the secondary wall as observed in other dicotyledonous plants (Harper and Bar-Peled, 2002). UDP-xylose is used for the backbone of xylans and its conversion from UDP-d-glucuronate is mediated by UDP-d-glucuronate carboxylase (UDP‐GlcA DCX). The peach UDP‐GlcA DCX was strongly over-expressed during endocarp lignification, while it remained at lower levels in the mesocarp (Hu et al., 2012).

## Future Perspectives

New investigations methods on the architectural and composition of cell walls, such as AFM and optical imaging approaches (for more detail see Sarkar et al., 2009), can aid in the understanding of cell wall modification occurring throughout the fruit patterning and development. This information is essential to correctly address the manipulation of the biosynthesis of primary metabolites used in cell wall building with the goal of rerouting them toward other biosynthetic pathways. Up to now, the most interesting advancements in this direction are regarding the manipulation of carbon flux for modifying cell wall polysaccharides composition and consequently fruit firmness and composition. A study has been carried out by silencing the tomato galacturonosyltransferase 4 (GAUT4), a member of enzyme family responsible for the pectin biosynthesis, showed that silenced fruits had an altered pectin composition, which coincided with an increase in firmness (De Godoy et al., 2013). Authors suggested that in silenced plants a shift in source to sink carbon partitioning occurred *via* the modulation of resource allocation *via* cell wall polysaccharides and raffinose metabolisms. For fruit trees, and in particular those harboring stone fruit, genetic transformation is still a long way for the difficulty to regenerate transformed plants (Prieto, 2011). However, it is possible by using agricultural practices to modify the carbon flux as demonstrated by covering with plastic film tangerine trees (Jin et al., 2018). Tangerine fruits collected from trees cultivated under plastic film were sweeter and softer. Authors suggested that the higher sugar accumulation in fruit may depend on the redistribution of carbohydrate toward fruit as indirectly supported by the parallel increase of sugar transporters gene expression in shaded trees. On the contrary, the modification of the water-soluble pectin and the protopectin content in shaded fruit resulted from the alteration of GAUTs and pectinesterases transcript profiles.

In conclusion, there are all premises for putting the reciprocal contribution between primary metabolism and cell wall into perspective to obtain better fruit as underlined by Beauvoit et al. (2018).

## Author Contributions

All authors have contributed significantly to the work and approved it for publication.

## Conflict of Interest

The authors declare that the research was conducted in the absence of any commercial or financial relationships that could be construed as a potential conflict of interest.
